# Oil Body-Bound Oleosin-rhFGF-10: A Novel Drug Delivery System that Improves Skin Penetration to Accelerate Wound Healing and Hair Growth in Mice

**DOI:** 10.3390/ijms18102177

**Published:** 2017-10-18

**Authors:** Wenqing Li, Jing Yang, Jingbo Cai, Hongyu Wang, Haishan Tian, Jian Huang, Weidong Qiang, Linbo Zhang, Haiyan Li, Xiaokun Li, Chao Jiang

**Affiliations:** 1 College of Life Sciences, Engineering Research Center of the Chinese Ministry of Education for Bioreactor and Pharmaceutical Development, Jilin Agricultural University, Changchun 130118, China; liwenqingshiwo@163.com (W.L.); yangjing5122010@163.com (J.Y.); cjb1045@163.com (J.C.); ccwhy@126.com (H.W.); hnjianhuang@yeah.net (J.H.); qiangweidong616@163.com (W.Q.); cczlb@126.com (L.Z.); 2 Wenzhou Biomedical Innovation Center, Wenzhou University, Wenzhou 325035, China; tianhaishan6418@163.com; 3 College of Life and Environmental Sciences, Wenzhou University, Wenzhou 325035, China

**Keywords:** oil body, oleosin-rhFGF-10, skin penetration, wound healing, hair growth

## Abstract

Recombinant human fibroblast growth factor 10 (rhFGF-10) is frequently used to treat patients with skin injuries. It can also promote hair growth. However, the effective application of rhFGF-10 is limited because of its poor stability and transdermal absorption. In this study, polymerase chain reaction (PCR) and Southern blotting were used to identify transgenic safflowers carrying a gene encoding an oleosin-rhFGF-10 fusion protein. The size and structural integrity of oleosin-rhFGF-10 in oil bodies extracted from transgenic safflower seeds was characterized by polyacrylamide gel electrophoresis and western blotting. Oil body extracts containing oleosin-rhFGF-10 were topically applied to mouse skin. The absorption of oleosin-rhFGF-10 was studied by immunohistochemistry. Its efficiency in promoting wound healing and hair regeneration were evaluated in full thickness wounds and hair growth assays. We identified a safflower line that carried the transgene and expressed a 45 kDa oleosin-rhFGF-10 protein. Oil body-bound oleosin-rhFGF-10 was absorbed by the skin with higher efficiency and speed compared with prokaryotically-expressed rhFGF-10. Oleosin-rhFGF-10 also enhanced wound closure and promoted hair growth better than rhFGF-10. The application of oleosin-rhFGF-10 in oil bodies promoted its delivery through the skin, providing a basis for improved therapeutic effects in enhancing wound healing and hair growth.

## 1. Introduction

Fibroblast growth factor 10 (FGF-10), also referred to as keratinocyte growth factor 2 (KGF-2), is a 24 kDa member of the fibroblast growth factor family and is composed of 208 amino acids [1]. Recombinant human FGF-10 (rhFGF-10) can enhance the proliferation of epidermal cells, and thereby promote the healing of injured skin [2]. It is frequently used to treat patients with mechanical injuries, burns and scalds of the skin, and it reduces scarring after healing [3]. Meanwhile, FGF-10 is necessary for hair growth, and may induce hair growth through activation of fibroblast growth factor receptor 2 [4], and also activation of the β-catenin and sonic hedgehog signaling pathways [5].

Although rhFGF-10 has been successful expressed in vitro [6,7], clinical application is limited because of its short half-life, poor thermal stability and low expression level. The heparin-binding domain of fibrin binds growth factors and can enhance the stability of FGF-10 and promote wound healing [8]. Polyethylene glycol modification of rhFGF-10 can decrease its immunogenic activity and improve its biostability, but its relative mitogenic activity is also reduced [9]. The oil body protein system has been utilized successfully to produce a variety of biologically active proteins in plant seeds, such as hirudin, insulin, epidermal growth factor and hyaluronidase [10,11,12,13,14]. In the process of seed maturation, 95% of the water content is lost [15], resulting in decreased activity of proteolytic enzymes, which improves the long-term storage of proteins and the effects of protein drugs. In light of the effects of FGF-10 in promoting cell division, proliferation, differentiation and migration, we have used the oil body protein system to produce an rhFGF-10 protein for delivery to skin. We used genetic engineering techniques to produce rhFGF-10 conjugated with the oil body-bound protein, oleosin. This is a novel approach to delivering drugs to skin, as the oil body not only enhances the stability of the recombinant protein, but also increases its skin penetration and subsequent effects on wound healing and hair growth.

## 2. Results

### 2.1. Stable Integration of the Oleosin-rhFGF-10 Gene in Safflowers

**Figure 1:**
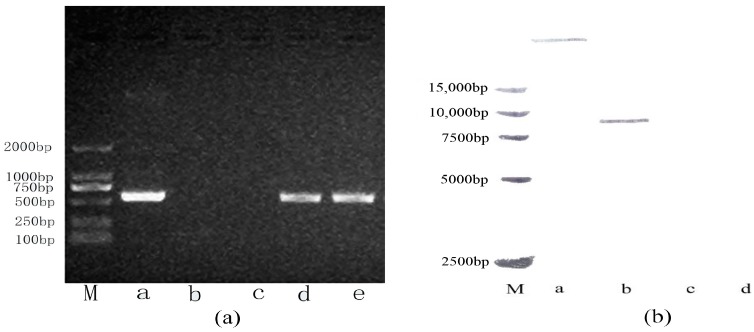
Integration of the oleosin-rhFGF-10 gene following safflower transformation. (**a**) Polymerase chain reactions (PCR) showing that the oleosin-rhFGF-10 gene had been inserted into the genome of transgenic safflowers. Lane M, molecular weight standards, lane a, positive control; lane b, negative control; lane c, blank control; lanes d–e, transgenic safflower lines. The PCR product is approximately 500 bp; (**b**) Southern blot showing a positive hybridization signal only for the positive control, lane a, and one of two transgenic safflower lines, lanes b–c. The wild type safflower, lane d, was negative. The number of oleosin-rhFGF-10 gene copies in the positive transgenic line was one.

A polymerase chain reaction (PCR) assay revealed that two lines of transgenic safflower carried the *oleosin-rhFGF-10* gene (Figure 1a). A PCR product of approximately 500 bp was amplified using rhFGF-10-specific primers, which was the expected size. A Southern blot analysis was performed to confirm that the oleosin-rhFGF-10 gene had integrated into the safflower genome of these lines, and to determine the copy number. Only one transformed line showed a hybridization signal, and the oleosin-rhFGF-10 copy number was one (Figure 1b). This transgenic safflower line was used for large-scale propagation, to provide experimental material for the rest of the study.

### 2.2. Oleosin-rhFGF-10 Protein Analysis

**Figure 2:**
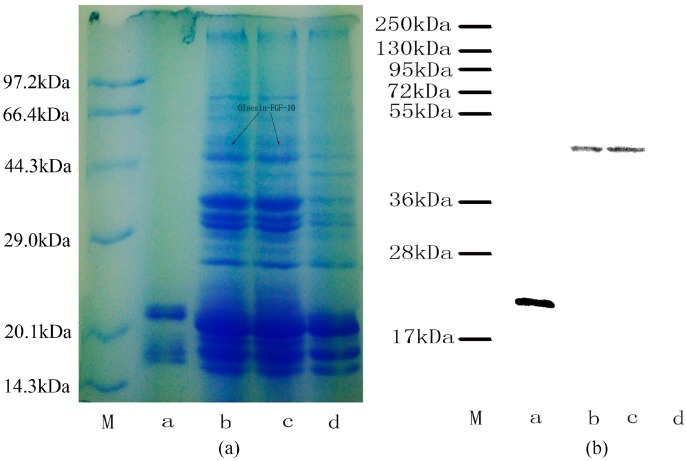
Protein analysis of oleosin-rhFGF-10. (**a**) Oil body proteins after 12% denaturing gel electrophoresis and Coomassie brilliant blue R250 staining. Lane M, molecular weight standards, lane a, rhFGF-10; lanes b–c, the same transgenic safflower lines; lane d, wild-type safflowers. Black arrows indicate the protein of interest and its molecular weight of 45 kDa; (**b**) western blot detection of the oleosin-rhFGF-10 protein. Lanes are as for (**a**).

The expression of oleosin-rhFGF-10 protein was confirmed by sodium dodecyl sulfate polyacrylamide gel electrophoresis and western blotting (Figure 2). The rhFGF-10 protein band was approximately 24 kDa and the oleosin-rhFGF-10 fusion protein band was approximately 45 kDa (Figure 2a). No band around 45 kDa was seen in wild-type (WT) oil body extract. Further western blotting experiments established that these proteins were rhFGF-10 and oleosin-rhFGF-10. The 24 kDa and 45 kDa bands stained positively for rhFGF-10 and oleosin-rhFGF-10 fusion protein, respectively (Figure 2b). The WT protein was negative. These results show that the protein of interest was successfully expressed in safflower seeds.

### 2.3. The Ability of Oleosin-rhFGF-10 to Penetrate the Skin

**Figure 3:**
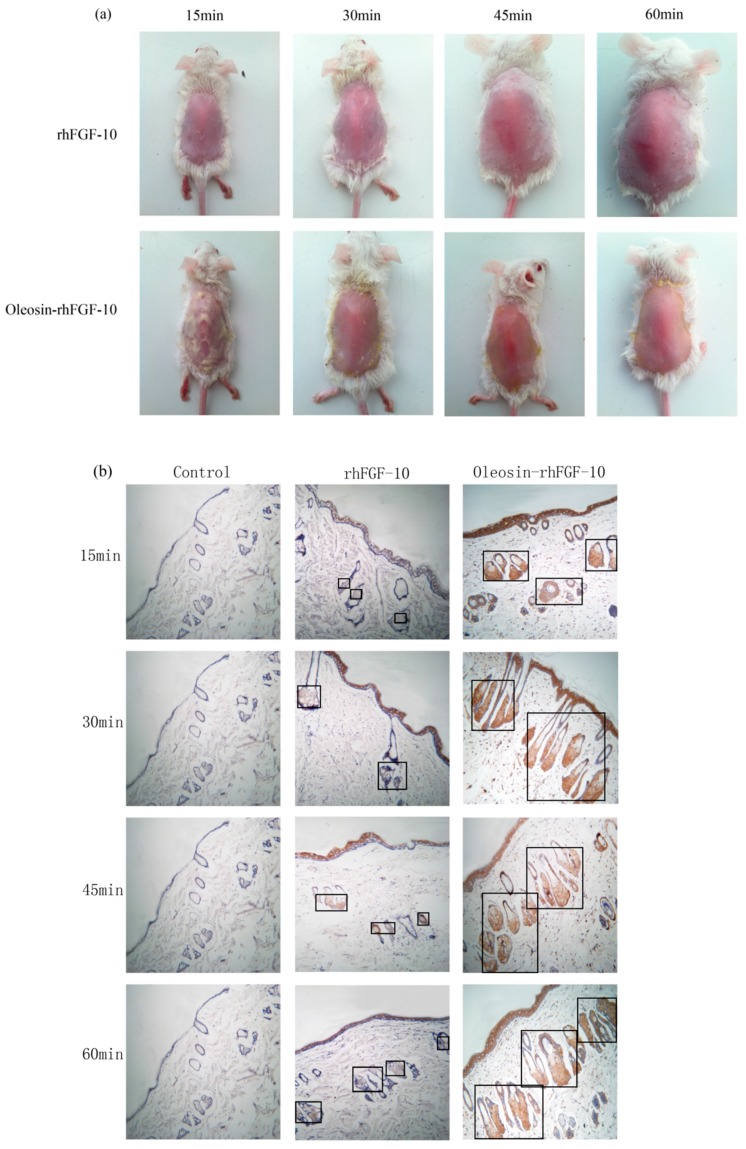

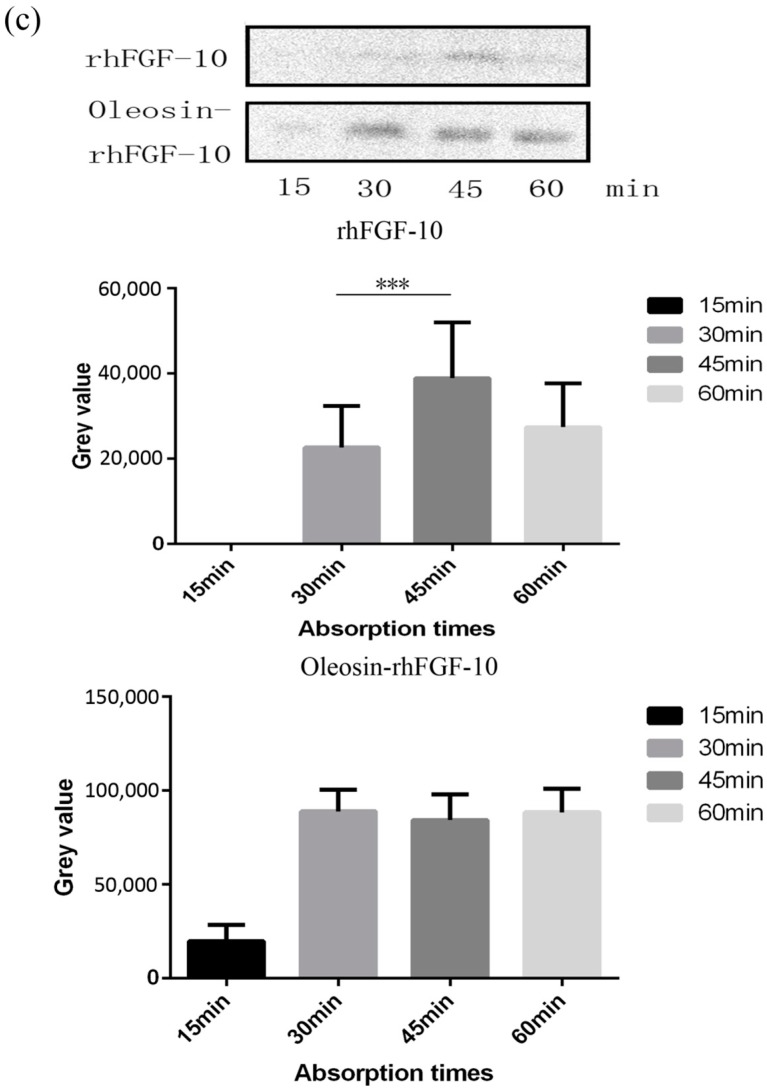
The ability of oleosin-rhFGF-10 to penetrate the skin. (**a**) The effectiveness of drug absorption into skin at different times, as evaluated by photography; (**b**) skin sections were stained for oleosin-rhFGF-10 by immunohistochemistry. The positive protein is stained brown and is marked by the black boxes; (**c**) the western blotting quantification method for objective evaluation in skin. The images are shown at 400× magnification. *** p < 0.001.

Recombinant proteins were applied to the skin and then detected by immunohistochemistry. First, we observed the absorption of the recombinant protein preparations into the surface of the skin. The absorption of prokaryotically-expressed rhFGF-10 was slow, and less of the preparation was absorbed; the surface of the skin in this group remained covered with a layer of protein which made the skin appear white. However, the absorption of oil body-bound oleosin-rhFGF-10 was better. After approximately 30 min, more than 90% of the preparation had been absorbed, and after 60 min, the preparation had been completely absorbed by the skin (Figure 3a). Next, immunohistochemistry was used to stain absorbed protein within the skin (Figure 3b). At times from 0 to 30 min, the positive protein was mainly concentrated in the epithelial cells. The staining intensity in the oleosin-rhFGF-10 group was greater than that in the rhFGF-10 group. More absorbed protein could be observed in the subepidermal tissue of the oleosin-rhFGF-10 group than that of the rhFGF-10 group, and its staining intensity was stronger. At times from 30 to 60 min, most of the protein had been absorbed, and was mainly concentrated in hair follicles, we observed the staining intensity had no significant change (Figure 3b). We analyzed the content of positive protein in skin by western blotting, and found that more positive protein was detected in the oleosin-rhFGF-10 group compared with the rhFGF-10 group (Figure 3c). But after 30 min, the positive protein was still being absorbed in the rhFGF-10 group, and the content of positive proteins reached maximum absorption at 45 min. In the oleosin-rhFGF-10 group, the content of positive proteins reached maximum absorption at 30 min, and the content of positive proteins remained stable. The stability of the proteins was possibly related. These data show that the oleosin-rhFGF-10 rapidly penetrated the skin and was absorbed faster than rhFGF-10.

### 2.4. Oleosin-rhFGF-10 Enhances Wound Closure

**Figure 4:**
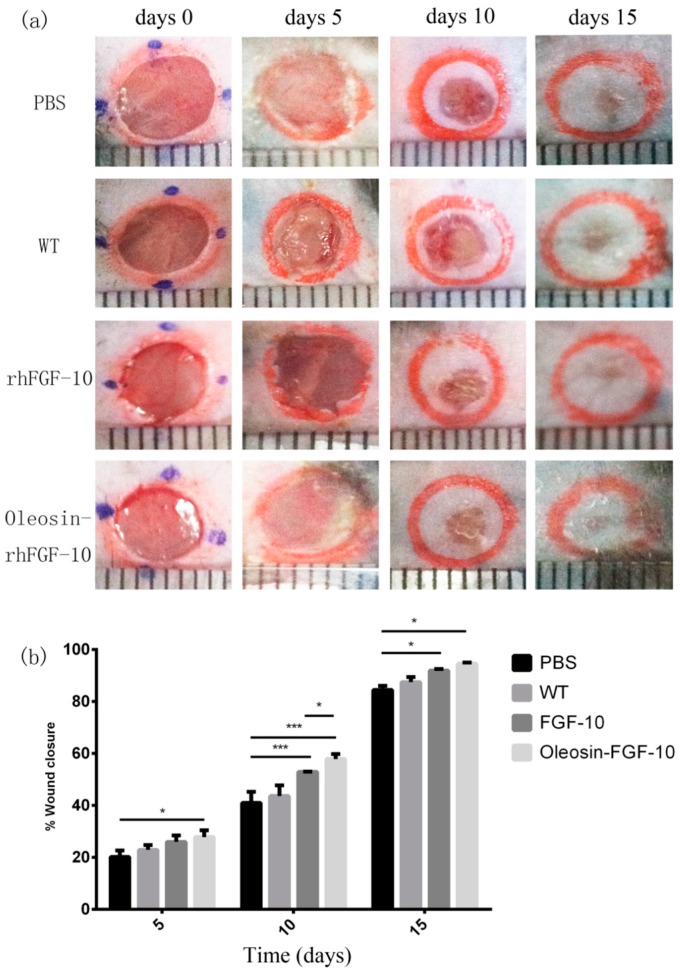
Oleosin-rhFGF-10 enhances wound closure. (**a**) Photographs of wounds receiving four treatments, on days 0, 5, 10 and 15. The scales shown at the bottom of each image are mm; (**b**) wound closure rates were quantified and statistically analyzed. Significant differences between the treatment groups (*n* = 6) were determined. *** *p* < 0.001, * *p* < 0.05.

To evaluate the bioactivity of oleosin-rhFGF-10 in promoting cutaneous wound healing, we created full-thickness cutaneous wounds in C57BL/6 mice. Figure 4a shows photographs of wounds in the four treatment groups on days 0, 5, 10 and 15. It can be seen that the phosphate-buffered saline (PBS) and WT oil body-treated wounds showed similar closure rates throughout the treatment process (*p* > 0.05). The wounds that were treated with rhFGF-10 protein and with an oil body extract containing oleosin-rhFGF-10 showed accelerated closure compared with the PBS and WT groups on days 10 (*p* < 0.001, *p* < 0.001) and 15 (*p* < 0.05, *p* < 0.05). On day 10, wounds treated with the oleosin-rhFGF-10 had closed more than those treated with rhFGF-10 (*p* < 0.05). After 15 days of oleosin-rhFGF-10 treatment, the surfaces of wounds were entirely healed and appeared almost scarless. The closure rates were compared among the four groups by quantitative analysis (Figure 4b). The wound closure rate in oleosin-rhFGF-10-treated mice was higher than the other three groups at all treatment times (days 5, 10 and 15). By day 15, the final wound closure rates were 94.5 ± 1.1% for the oleosin-rhFGF-10 group, 91.9 ± 1.4% for the rhFGF-10 group, 87.4 ± 4.4% for the WT group and 84.5 ± 3.5% for the PBS group.

### 2.5. Oleosin-rhFGF-10 Induces More Subcutaneous Tissue Formation

**Figure 5:**
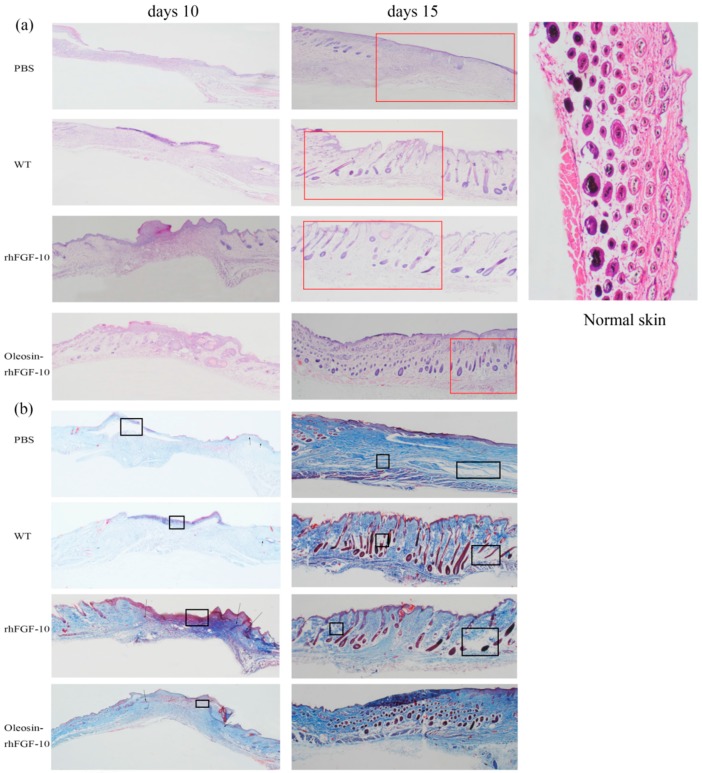
(**a**) Histological analysis of wound sites. Sections of wound skin at two time points following four treatments were stained with hematoxylin and eosin. The images are shown at 40× magnification; (**b**) collagen deposition in wound sites. Sections of wound skin at two time points following four treatments were stained with Masson’s trichrome. The unrepaired parts were marked by the black boxes and repair parts are marked by the arrows. The wound areas were marked by red boxes. The images are shown at 40× magnification.

For clinical observation and histological analysis of skin regeneration, mice were randomly selected from each group and euthanized on days 10 and 15. Figure 5a shows the hematoxylin and eosin (H&E) stained histology for the four treatment groups. On day 10, in rhFGF-10 and oleosin-rhFGF1-10 groups, the wound areas had begun to close and we could observe the regeneration of the epidermis. On the other hand, regeneration was not complete in the rhFGF-10 groups, which showed fewer hair follicles and other skin appendages. On day 15, the wounds had failed to heal perfectly in the PBS and WT groups, but in the oleosin-rhFGF-10 and rhFGF-10-treated groups, complete regeneration of the skin structure was seen. However, the hair follicles and blood vessels had regenerated and were well-integrated with surrounding regions. The connectives tissue was dyed red in the oleosin-rhFGF-10 group deeper than in the rhFGF-10 group.

Sections of wounds were also stained using Masson’s trichome to evaluate collagen deposition in the regenerating skin. On day 10, wounds treated with oleosin-rhFGF-10 showed much greater collagen deposition and appendage density than the other three groups (Figure 5b). At day 15, the extent of collagen deposition and thickness of the collagen layer in the subepidermal wound tissue made the skin structure more complete, especially in the vicinity of the epidermis. In contrast, less collagen formation was observed in the PBS and WT groups, and the dense degree of collagen was lost. Similar results were seen with the H&E staining, shown in Figure 5. Thus, the oleosin-rhFGF-10 accelerated wound healing by increasing reepithelialization, subepidermaltissue formation and collagen deposition.

### 2.6. Oleosin-rhFGF-10 Promotescytokeratin-10 and IL-2 Expression

**Figure 6:**
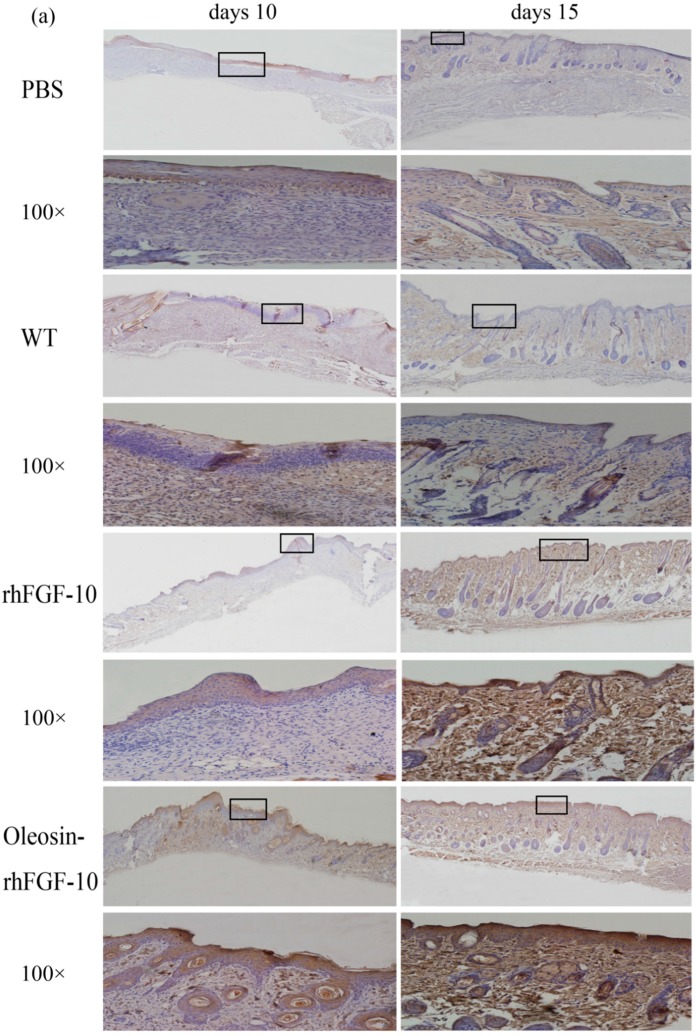

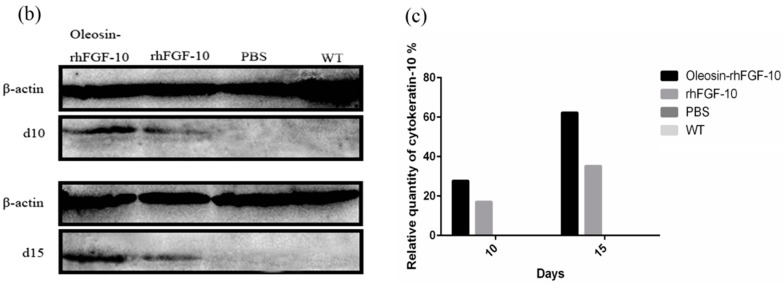
(**a**) Immunohistochemical assay with thecytokeratin-10 in wounds of the four groups. The cytokeratin-10 was detected in regenerated epidermis. The images are shown at 40× and 100× magnification; (**b**,**c**) western blot quantification method for objective evaluation for expressing cytokeratin-10 in regenerated skin.

**Figure 7:**
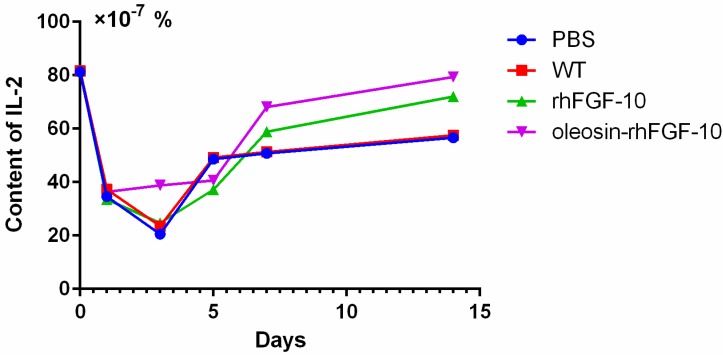
IL-2 was measured by enzyme-linked immunosorbent assay (ELISA) in the process of wound healing. Mice were randomly selected from each group and euthanized on days 1, 3, 5, 7 and 14. The entire experiment was repeated three times and results were expressed through a mean.

Cytokeratin-10 was further used as a stained mark to evaluate re-epithelialization. Consistent with the HE and Masson results, the epidermis regeneration of skin wounds were not, or were least, observed in both PBS and the WT at 10 and 15 days; and the oleosin-rhFGF-10 treated wounds showed much thicker epithelia layers than rhFGF-10 treated wounds from days 10 to 15. Meanwhile, the number of skin appendages in the oleosin-rhFGF-10 group was more than the other three groups (Figure 6a). From days 1 to 3, the content of IL-2 (Interleukin-2) was in a downward trend in the PBS and WT rhFGF-10 groups; but in the oleosin-rhFGF-10 group the content of IL-2 was on the rise. From days 5 to 14, the content of IL-2 was on the rise; at day 14, IL-2 in the rhFGF-10 (72.0%) and oleosin-rhFGF-10 (79.4%) groups was higher than in the PBS (56.6%) and WT (57.6%) groups, which were closer to the content of IL-2 (81.2%) in normal mice (Figure 7).

Taken together, the oleosin-rhFGF-10 exhibited an improved healing effect on re-epithelialization, granulation tissue formation, and collagen deposition. IL-2 levels in the body in general have the potential to impact the process of wound healing. So when the skin is damaged at days 1 to 3, the oleosin-rhFGF-10 can sustain high levels of the IL-2 in order to promote wound healing. At day 14, the levels of IL-2 in the oleosin-rhFGF-10 group was higher than other three groups, and it promoted wound healing better than other groups.

### 2.7. The Effect of Oleosin-rhFGF-10 on Hair Growth

**Figure 8:**
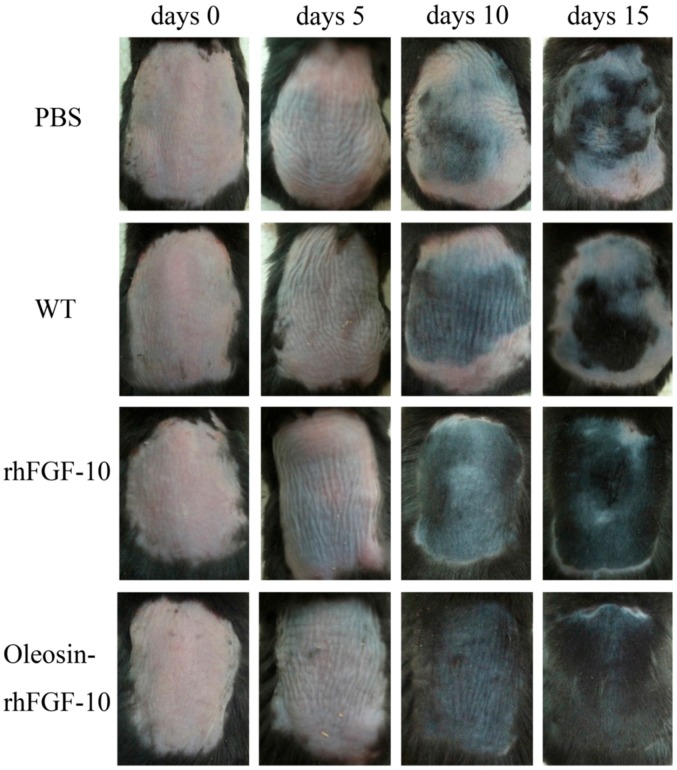
The effect of oleosin-rhFGF-10 on hair growth. Sequential photographs showing hair growth following four treatments, on days 0, 5, 10 and 15. The color of the skin changes from pink to black with the regeneration of hair follicles.

To test the hair growth activity of oleosin-rhFGF-10, we applied PBS, WT oleosin, rhFGF-10 and oleosin-rhFGF-10 to a C57BL/6 mouse alopecia model for 15 days. Every 5 days, we observed shades of color on the dorsum to evaluate the extent of hair growth (Figure 8). At day 10, the oleosin-rhFGF-10 group showed the blackest coloration of the groups, and a few newly emerging hairs could be observed. In contrast, less hair growth was seen in the PBS and WT groups, which was associated with less black coloration. At day 15, the rhFGF-10 and oleosin-rhFGF-10 groups showed dense hair growth, and their hair coverage rate was over 90%, compared with coverage rates of less than 50% in the PBS and WT groups.

**Figure 9:**
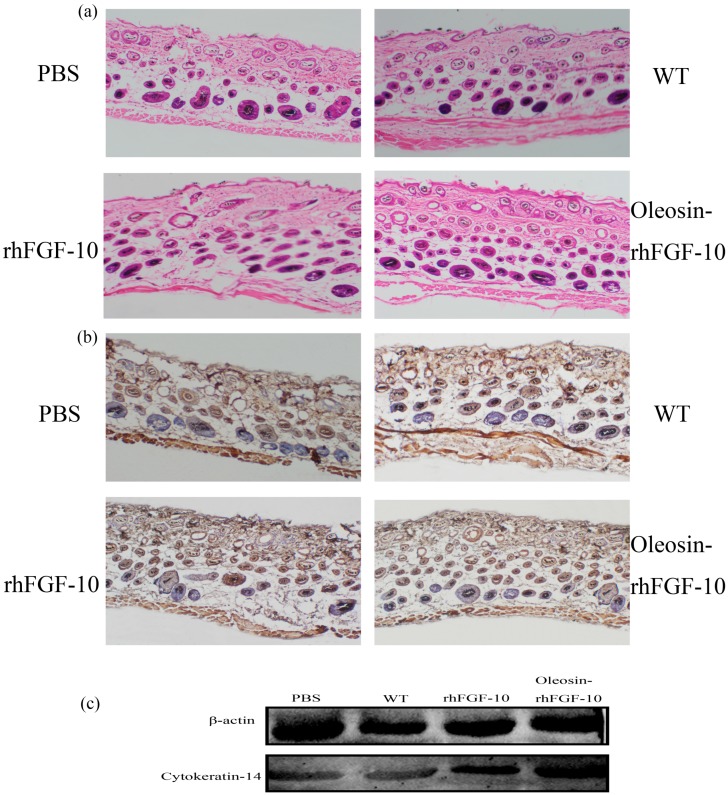
The effect of oleosin-rhFGF-10 on hair-follicle regeneration. (**a**) Images of hematoxylin- and eosin-stained skin sections following four treatments, showing hair regeneration on day 10. Images are shown at 100× magnification; (**b**) immunohistochemistry of the expression ofcytokeratin-14; (**c**) western blot quantification method for objective evaluation for expressing cytokeratin-14 in skin.

**Figure 10:**
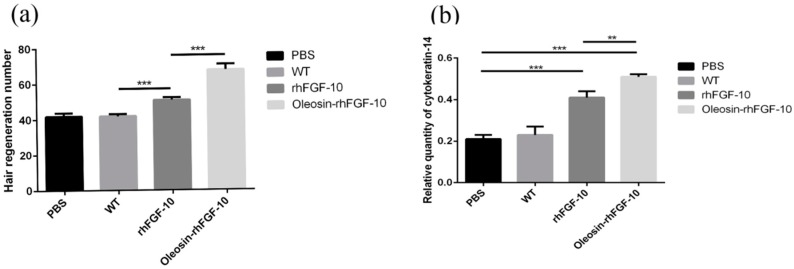
The number of regenerating hair follicles and expressing cytokeratin-14 were quantified. (**a**) Presenting the number of hair follicles; (**b**) the relative content ofcytokeratin-14. *** *p* < 0.001, **p < 0.05.

We observed hair regeneration in H&E-stained skin sections. As shown in Figure 9a, the number of hairs regenerating in the rhFGF-10 and oleosin-rhFGF-10 groups was higher than in the other groups; and the length of hair follicles was greater in the oleosin-rhFGF-10 group compared with the other three groups, which was consistent with the results in Figure 8. Quantitatively, more hair regeneration was seen following treatment with oleosin-rhFGF-10 compared with rhFGF-10, WT oil body protein and PBS (*p* < 0.05, *p* < 0.001, *p* < 0.001) (Figure 10a). To evaluate the role of cytokeratin-14 induction in the mechanism of hair growth, cytokeratin-14 expression was detected by immunohistochemistry. The protein of interest was expressed in all four treatment groups, but the levels of cytokeratin-14 were higher in the oleosin-rhFGF-10 group compared with the other three groups, with much of the positive protein localized in the hair follicles (Figure 9b and Figure 10b).

### 2.8. Stability of Oleosin-rhFGF-10

**Figure 11:**
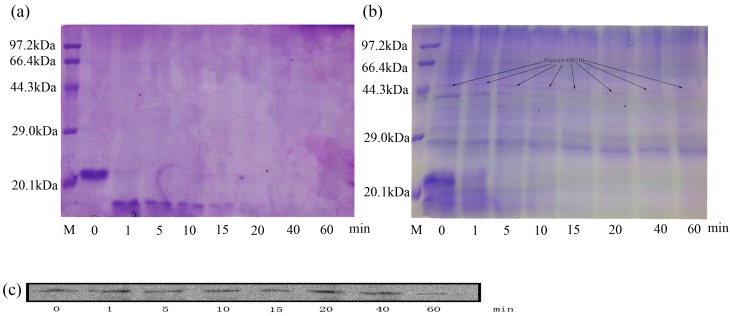
Sodium dodecyl sulfate polyacrylamide gel electrophoresis (SDS-PAGE) and western blot to analyze the proteolysis. (**a**) The method of SDS-PAGE to analyze the rhFGF-10; (**b**,**c**) the oleosin-rhFGF-10 was detected by SDS-PAGE and western blot to confirm the stability of interest protein.

To detect the potential resistance of oleosin-rhFGF-10 to proteolysis, the rhFGF-10 and oleosin-rhFGF-10 protein were incubated with trypsin for various time periods and then analyzed by sodium dodecyl sulfate polyacrylamide gel electrophoresis (SDS-PAGE) and western blotting. Figure 11a shows that, after incubation for 1 min, rhFGF-10 had been degraded, and appreciable change was seen in the molecular size and intensity of the band. At 40 min, rhFGF-10 had been fully digested. However, for oleosin-rhFGF-10, no significant degradation was observed from 1 to 60 min, and there was no tendency for the band to change position (Figure 11b,c). However, proteins with a molecular weight of less than or equal to 23 kDa were digested. These results demonstrated that oleosin-rhFGF-10 is a stable protein and resistant to proteolysis.

## 3. Discussion

FGF-10 has been shown to effectively promote epidermal regeneration, corneal wound healing, lung wound healing and hair growth [2,3,5,16,17]. The shortcomings of rhFGF-10 include its instability, poor skin absorption and short half-life. To overcome these disadvantages, we transferred an rhFGF-10 gene into safflowers to mediate expression of a fusion protein in their seeds. The pharmacological activity and advantages of this fusion protein were experimentally validated. First of all, the oleosin-rhFGF-10 was used in a transdermal absorption test. The immunohistochemical results showed that the fusion protein better penetrated into the skin compared with unmodified rhFGF-10. The oleosin-rhFGF-10 preparation likely preserved the oil body structure, which mainly consists of three parts: the outer layer of phospholipids, abundant triacylglycerol, and oleosin protein, which is localized perpendicular to the surface of oil bodies and is responsible for the stability of the oil body [18]. Furthermore, the permeability barrier of skin, the stratum corneum, mostly consists of saturated lipids such as ceramide, cholesterol and free fatty acids. Oil bodies have a similar chemical composition, particularly with respect to fatty acids, which can improve their skin penetration ability [19]. The oil body integrates into the stratum corneum, leading to changes in lipid composition and structure, along with the appearance of cracks and holes on the skin surface that increase the permeability of skin and allow drugs to be more readily absorbed [20]. On the other hand, hair follicles and sebaceous glands are another important route of transdermal drug absorption [21]. In our study, the oil body protein appeared to be absorbed via the hair follicle-sebaceous gland route. The oil body-derived fusion domain of oleosin-rhFGF-10 likely increased fat-solubility and thereby improved its absorption via this route. As shown in Figure 3b, a large amount of oleosin-FGF-10 protein localized in hair follicles, and the intensity of staining was higher than for the other treatment groups. The large amount of positive protein concentrated in hair follicles is probably an important reason why oleosin-rhFGF-10 promoted hair growth better than the other treatments. Meanwhile, the oleosin-rhFGF-10 fusion protein showed increased resistance to proteolysis due to enhanced protein stability. The larger size of the fusion protein would raise its molecular volume, leading to reduced proteolytic enzyme-binding capacity, thereby protecting the protein from trypsin attack. Consistent with this, the results in Figure 11b show that proteins of less than or equal to 23 kDa are easier for trypsin to digest. Moreover, it is possible that oleosin-rhFGF-10 bound on the surface of the oil body leads to aggregation in an insoluble form and thus cannot be degraded by trypsin; but when it is used as a topical application on skin, the structure of the oil body was destroyed and the oleosin-rhFGF-10 was released, so the oleosin-rhFGF-10 restored solubility so that it can penetrate skin to perform bioactivities.

One study has reported that the blood IL-2 levels in burn patients were increased on both day 1 and day 5, and it can promote wound healing [22]. From days 1 to 3 and days 7 to 15, the oleosin-rhFGF-10 can make the IL-2 sustain high levels, which can promote wound healing better than other three groups. It is also possible that the oleosin-rhFGF-10 had better stability, so it can work better, lasts longer, and a strong immune response could to reduce the wound infection. At day 5, in the rhFGF-10 and oleosin-rhFGF-10 groups, the expression of IL-2 was lower than in the PBS and WT groups. As the wound begins to close and tissue started to regenerate, the strong immune response could be harmful and damage wound healing. At day 7 and onwards, when the wound had healed more, the IL-2 could promote wound healing rather than damaging it.

FGF-10 has been found in dermal papilla fibroblasts and its receptor, fibroblast growth factor receptor 2 (FGFR2), in the neighboring outer root sheath of keratinocytes. Application of rhFGF-10 at 10 ng/mL significantly stimulated human hair-follicle cell proliferation in organ culture (26–35%) [4]. In this study, we created full-thickness cutaneous wounds in mice, which led to loss of hair follicles in the wound area. When we applied the oil body protein containing the oleosin-rhFGF-10 to treat the wounds, we found better curative effect in the oleosin-rhFGF-10 group than in the rhFGF-10 group, especially in promoting hair-follicle regeneration; meanwhile, the hair follicles regenerated made the wound healing more complete and the skin structure closer to the normal skin, reflecting another advantage of oleosin-rhFGF-10 in treating wound healing. Using the oil body as a drug delivery system could promote more oleosin-rhFGF-10 being absorbed in healed skin, hence more regenerated hair follicles in the oleosin-rhFGF-10 group than in the other three groups, and this may be relative to the stability of oleosin-rhFGF-10. For example, when applied in the treatment of head skin wounds, it might not only speed up wound healing, but also it could promote hair growth more quickly.

More generally, oil bodies as a delivery system can promote transdermal drug absorption, ferrying large molecules such as proteins into the skin and accelerating their uptake. They provide a new approach to solving the problems associated with inducing macromolecular protein drugs to cross the skin barrier. Furthermore, transgenic safflowers provide a new way to produce valuable medicinal proteins, in this case oleosin-rhFGF-10. The oleosin-rhFGF-10 effectively accelerated wound healing and hair growth in mice, suggesting it could be a possible new drug for the treatment of skin wounds and hair loss in humans. Finasteride and minoxidil are internationally approved for the treatment of hair loss, but their curative effects are unstable and they have side effects, which limits their more extensive use. Our oil body-oleosin-rhFGF-10 preparation not only efficiently promoted hair growth, but is also a natural product. There may be less toxicity and side effects of oil body proteins compared with synthetic drugs. Natural and green products developed as new drugs have good potential for application.

## 4. Materials and Methods

### 4.1. Polymerase Chain Reaction (PCR) and Southern Blot Detection of the Oleosin-rhFGF-10 Gene in Safflower

The T3 generation transgenic safflower used in this study was obtained from Jilin Agricultural University, China. The cetyl trimethylammonium bromide method was used to isolate genomic DNA from transgenic plants. PCR and Southern blotting were carried out using genomic DNA from T3 plants, with a pOTBar-oleosin-rhFGF10 plasmid (Jilin Agricultural University, Jilin, China) used as the positive control, WT safflower leaf DNA as the negative control, and double distilled water as the blank control. The PCR reaction program was: denaturation at 95 °C for 5 min; 30 cycles of 95 °C for 30 s, 55 °C for 30 s, 72 °C for 1 min; and a final extension at 72 °C for 10 min. The rhFGF-10-specific primers used were: forward, 5′-ATAGGTACGCAACGGGAGA-3′; reverse, 5′-TGCCTTCACAGCGACAAC-3′. The PCR products were characterized by 1% agarose gel electrophoresis. The identification of transgenic safflower genomes was verified by Southern blotting. A digoxigenin-UTP probe was synthesized using the pOTBar-oleosin-rhFGF10 plasmid as a template, with a DIG High Prime DNA Labeling and Detection Starter Kit I (DIG High Prime DNA Labeling and Detection Starter Kit I (for color detection with NBT/BCIP), 11585614910, Basel, Switzerland). The probe DNA was separated on a 1% agarose gel electrophoresis. It was then purified with a gel extraction kit (Biotake, Changchun, China). The genomic DNA was digested with *HindIII* and subjected to 0.8% agarose gel electrophoresis at 38 V for 11 h. The gel was then placed in denaturing solution to denature the DNA, and the DNA was transferred to a nylon membrane by capillary blotting. The membrane was prehybridized at 43 °C for 2 h and hybridized for 10 h. The membrane was then incubated in blocking buffer at room temperature for 30 min. Next, the membrane was incubated with anti-Dig-AP (1:5000) at 25 °C for 30 min, washed, and equilibrated in detection buffer for 5 min. Finally, the membrane was incubated in nitro blue tetrazolium chloride/5-bromo-4-chloro-3-indolyl phosphate stock solution for 30 min and photographed to record the hybridization signals.

### 4.2. Identification of the Fusion Protein

Seeds harvested from transgenic safflowers (0.5 g) were crushed and ground in 1000 μL of 0.01 M phosphate buffered saline (PBS, pH 7.4) using a mortar and pestle, and then centrifuged for 10 min at 12,000× *g*. The aqueous phase was discarded and the hydrophobic phase was washed three times with PBS and centrifugation, after which the hydrophobic supernatant containing oil body protein material was collected. The transgenic safflowers oil body protein was a mix protein which contained the oleosin-rhFGF-10 protein, other oleosin and oil body; and the rhFGF-10 protein was highly purified protein (expressed in *E. coli*, supplied by the Wenzhou Medical University, Wenzhou, China); the wild-type safflower oil body protein only contained oleosin and oil body (supplied by the Jilin Agricultural University). Extracted oil body protein was denatured and the oil body broken up by heating, separated on 12% polyacrylamide electrophoresis gels and western blotted onto PVDF membranes. The membrane was blocked in 5% skim milk for 2 h, then incubated with poly-clonal rabbit anti-rhFGF-10 (ab71794, 1:1000, Abcam, Shanghai, China) at 4 °C overnight. The membrane was washed three times with PBS–tween 20 (PBST), 10 min per wash. Next, the membrane was incubated with horse radish peroxidase (HRP) conjugated goat anti-rabbit antibody (bs-0295G-HRP, 1:1000, Bioss, Beijing, China) at room temperature for 1 h and washed in PBST as above. Signals were detected by enhanced chemiluminescence. The concentration of oleosin-rhFGF-10 was determined using a human KGF-2 enzyme-linked immunosorbent assay (ELISA) kit (Baijin Biotechnology Co., Ltd., Changchun, China).

### 4.3. Mouse Model

C57BL/6 (20 ± 2 g) and BALB/c mice (20 ± 2 g) were purchased from Changchun Billion Biotechnology Limited Company (Changchun, China). The mice were housed in normal laboratory conditions (temperature, 23 ± 2 °C, humidity, 40–60%, photoperiod, 12 h light and 12 h darkness) with free access to a standard diet and water. The animals were allowed to adjust to their new environment for one week. The investigation conforms with the Guide for the Care and Use of Laboratory Animals published by the US National Institutes of Health (NIH Publication No. 85-23, revised 1996). The experiment was authorized by Jilin Agricultural University ethical committee.

### 4.4. The Ability of Oil Body to Promote the Oleosin-rhFGF-10 to Penetrate the Skin of Mice

Healthy male BALB/c mice were randomly divided into three groups (*n* = 6 per group) and anesthetized with 4% chloral hydrate (0.02 mL/g). Hair was removed from their dorsum. The PBS group was treated with 0.01 M PBS (pH 7.4), the rhFGF-10 group with 0.2 μg/μL rhFGF-10 [23], and the oleosin-rhFGF-10 group with 143 μg/μL oil body extract containing oleosin-rhFGF-10 at 0.2 μg/μL. After 15, 30, 45 or 60 min, the mice were euthanized by cervical dislocation. The dorsal skin was photographed, and then processed for immunohistochemical staining to detect rhFGF-10 and oleosin-rhFGF-10 protein. The rest of the skin tissues were analyzedby western blotting. The total proteins were extracted with a Total Protein Extraction Kit (Solarbio, Beijing, China). Separated on 12% polyacrylamide electrophoresis gels and western blotted onto PVDF membranes, 30 μg protein was used to analyze each sample. The Quantity One software (Bio-Rad, Hercules, CA, USA) was used to analyze the gray degree values of the signals.

### 4.5. Effect of Oleosin-FGF-10 on Mouse Skin Wound Healing

Healthy male C57BL/6 mice were randomly divided into four groups (*n* = 6 per group). The mice were anesthetized by intraperitoneal injection of 4% chloral hydrate (0.02 mL/g). Hair was shaved and then a depilatory cream was evenly applied on the dorsum, which was then cleansed with 75% ethanol for surface sterilization. Two full-thickness cutaneous wounds were made with a scalpel, one on either side of the spine. The PBS group was treated with0.01 M PBS (pH 7.4); the WT group with 357 mg/L oil body extract; the rhFGF-10 group with 0.5 mg/L rhFGF-10 [24]; and the oleosin-rhFGF-10 group with 357 mg/L oil body extract containing oleosin-rhFGF-10 at 0.5 mg/L. ATegaderm™ transparent dressing (3M Health Care, Brookings, Washington, DC, USA) was then applied to the wounds to avoid infections and keep the surface of the wound from becoming wet. These treatments were repeated once every day. Mice were kept in separate cages. Photographs of the wound site at different time points were used to calculate the wound closure rate by using Image-Pro Plus 6.0 (Media Cybernetics, Inc., Rockville, MD, USA) software to trace the wound margin.

On the tenth day after wounding, three mice were randomly selected from every group and euthanized; the remaining mice were euthanized on day 15. The regeneration of wounds and surrounding tissues was examined histologically. Wound closure was quantified according to the following equation.

$$\mathrm{Wound}\;\mathrm{closure}=\frac{\;\mathrm{area}\;\mathrm{of}\;\mathrm{original}\;\mathrm{wound}-\mathrm{area}\;\mathrm{of}\;\mathrm{acture}\;\mathrm{wound}\;}{\mathrm{area}\;\mathrm{of}\;\mathrm{original}\;\mathrm{wound}}\times 100\%$$

### 4.6. IL-2 Enzyme-Linked Immunosorbent Assay (ELISA)

ELISA of IL-2 was performed using the Mouse IL-2 ELISA Kit (bsk00030, Bioss, Beijing, China) and following the manufacturer’s instructions.

### 4.7. Effect of Oleosin-rhFGF-10 on Hair Growth

Healthy male C57BL/6 mice were randomly divided into four groups (*n* = 6 per group). The mice were anesthetized by intraperitoneal injection of 4% chloral hydrate (0.02 mL/g). Hair on the dorsum was shaved with electric clippers, and a hair removal cream was used to remove subcutaneous hairs to establish an alopecia model. The PBS group was given 0.01 M PBS (pH 7.4); the WT group was given 7.14 mg/L oil body extract; the rhFGF-10 group was given 0.01 mg/l rhFGF-10 [4]; and the oleosin-rhFGF-10 group was given 7.14 mg/L oil body extract containing oleosin-rhFGF-10 at 0.01 mg/L. After a 30 min wait to allow for the drug to fully absorb, the mice were returned to their cages. The treatment was repeated once every day. The condition of hair growth on the dorsum of the mice was observed and photographed. The mice were euthanized on day 10, and skin samples were taken for histological analysis of hair regeneration. From each treatment group, six representative histological sections were randomly selected for quantitative analysis of the number of regenerating of hair follicles. Differences between groups were compared by statistical analysis. Cytokeratin-14 expression in similar sections was evaluated by immunohistochemistry and western blot.

### 4.8. Effect of Oleosin on Enzymatic Hydrolysis of rhFGF-10

rhFGF-10 and oleosin-rhFGF-10 were compared, each at eight time points. PBS (100 μL, 0.01 M, pH 7.4) was added to 50 μg rhFGF-10 or 50 μg total oleosin including oleosin-rhFGF-10. The rhFGF-10 and oleosin-rhFGF-10 were incubated with trypsin (2 mM) in a volume ratio of 100:1 protein: trypsin at 37 °C for the times indicated. Next, 5× loading buffer was added and the protein and trypsin were denatured at 100 °C for 10 min. After completing them, the samples were then subjected to sodium dodecyl sulfate polyacrylamide gel electrophoresis (SDS-PAGE) and western blot to evaluate the degradation of protein.

### 4.9. Histological Analysis

Skin samples were 1 cm × 1 cm × 0.15 cm, and were fixed in 0.01 M PBS (pH 7.4) containing 4% paraformaldehyde for 8 h and then routinely processed to wax. Sections were cut at 5 μm and adhered to poly-l-lysine coated glass slides. The histology of subcutaneous tissues was analyzed with H&E staining (Solarbio, Beijing, China), or with Masson’s trichrome staining (Solarbio, Beijing, China) for the evaluation of collagen content in healing wounds.

### 4.10. Immunohistochemical Staining

Sections were dewaxed, hydrated and pretreated with 3% H_2_O_2_ for 15 min to quench endogenous peroxidase activity, and then washed three times with PBS, 5 min per wash. Antigen retrieval was performed at high temperature and pressure in 0.01 Msodium citrate buffer for 10 min. Nonspecific antibody binding was blocked with PBS containing 5% bovine serum for 60 min at room temperature. Next, the sections were incubated with the primary antibody, either rabbit polyclonal anti-FGF-10 (sc-7179, 1:100, Santa Cruz Biotech, Santa Cruz, CA, USA); rabbit polyclonal anti-cytokeratin-10 (bs-11186R, 1:200, Bioss, Beijing, China); or rabbit polyclonal anti-cytokeratin-14 (bs-17922R, 1:250, Bioss, Beijing, China), at 4 °C overnight, and then with the secondary antibody, HRP-conjugated goat anti-rabbit (bs-0295G-HRP, 1:1000, Bioss, Beijing, China), for 2 h at room temperature. Antibody binding was detected with a DAB chromogen kit (Solarbio, Beijing, China) and the sections were counter stained with H&E.

### 4.11. Statistical Analysis

Data are expressed as the mean ± standard deviation. Data were analyzed using one-way analysis of variance followed by Tukey’s test, using GraphPad Prism 5 software (GraphPad Software Inc., La Jolla, CA, USA).

## Abbreviations

PCR	Polymerase Chain Reaction
SDS-PAGE	Sodium dodecyl sulfate polyacrylamide gel
PBS	Phosphate buffer salt
WT	Wild Type
rhFGF-10	Recombinant human fibroblast growth factor 10
Oleosin-rhFGF-10	Oleosin-Recombinant human fibroblast growth factor 10
ELISA	Enzyme-Linked Immunosorbent Assay
DIG	Digoxigenin
NBT/BCIP	Nitroblue Tetrazolium/5-Bromo-4-Chloro-3-Indolyl Phosphate
AP	Alkaline Phosphatase
HRP	Horse radish peroxidase
DAB	Diaminobenzidine
PVDF	Polyvinylidene Fluoride
